# Collection of human and environmental data on pesticide use in Europe and Argentina: Field study protocol for the SPRINT project

**DOI:** 10.1371/journal.pone.0259748

**Published:** 2021-11-15

**Authors:** Vera Silva, Abdallah Alaoui, Vivi Schlünssen, Anne Vested, Martien Graumans, Maurice van Dael, Marco Trevisan, Nicoleta Suciu, Hans Mol, Karsten Beekmann, Daniel Figueiredo, Paula Harkes, Jakub Hofman, Ellen Kandeler, Nelson Abrantes, Isabel Campos, María Ángeles Martínez, Joana Luísa Pereira, Dirk Goossens, Juergen Gandrass, Freya Debler, Esperanza Huerta Lwanga, Marlot Jonker, Frank van Langevelde, Martin T. Sorensen, Jerry M. Wells, Jos Boekhorst, Anke Huss, Daniele Mandrioli, Daria Sgargi, Paul Nathanail, Judith Nathanail, Lucius Tamm, Peter Fantke, Jennifer Mark, Christian Grovermann, Ana Frelih-Larsen, Irina Herb, Charlotte-Anne Chivers, Jane Mills, Francisco Alcon, Josefina Contreras, Isabelle Baldi, Igor Pasković, Glavan Matjaz, Trine Norgaard, Virginia Aparicio, Coen J. Ritsema, Violette Geissen, Paul T. J. Scheepers

**Affiliations:** 1 Soil Physics and Land Management Group, Wageningen University & Research, Wageningen, Netherlands; 2 Institute of Geography, University of Bern, Bern, Switzerland; 3 Centre for Development and Environment, University of Bern, Bern, Switzerland; 4 Department of Public Health, Aarhus University, Aarhus, Denmark; 5 National Research Centre for the Working Environment, Copenhagen, Denmark; 6 Radboud Institute for Health Sciences, Radboudumc, Nijmegen, Netherlands; 7 Department for Sustainable Food Process (DISTAS), Università Cattolica del Sacro Cuore, Piacenza, Italy; 8 Wageningen Food Safety Research, Wageningen, Wageningen University & Research, Wageningen, Netherlands; 9 Institute for Risk Assessment Sciences, Utrecht University, Utrecht, Netherlands; 10 Research Centre for Toxic Compounds in the Environment (RECETOX), Faculty of Science, Masaryk University, Brno, Czech Republic; 11 Institute of Soil Science and Land Evaluation, Soil Biology Department, University of Hohenheim, Stuttgart, Germany; 12 Centre for Environmental and Marine Studies and Department of Environment and Planning, University of Aveiro, Aveiro, Portugal; 13 Centro de Investigaciones Energéticas, Medioambientales y Tecnológicas–CIEMAT, Madrid, Spain; 14 Centre for Environmental and Marine Studies and Department of Biology, University of Aveiro, Aveiro, Portugal; 15 KU Leuven Department of Earth and Environmental Sciences, Geo-institute, Celestijnenlaan, Leuven, Belgium; 16 Institute of Coastal Environmental Chemistry, Organic Environmental Chemistry, Helmholtz-Zentrum Hereon, Geesthacht, Germany; 17 Dutch Mammal Society, Nijmegen, Netherlands; 18 Wildlife Ecology and Conservation Group, Wageningen University & Research, Wageningen, Netherlands; 19 Department of Animal Science, Aarhus University, Aarhus, Denmark; 20 Host-Microbe Interactomics, Animal Sciences Group, Wageningen University & Research, Wageningen, Netherlands; 21 Cesare Maltoni Cancer Research Center, Ramazzini Institute, Bologna, Italy; 22 Land Quality Management—LQM, Nottingham, United Kingdom; 23 Research Institute of Organic Agriculture—FIBL, Frick, Switzerland; 24 Quantitative Sustainability Assessment, Department of Technology, Management and Economics, Technical University of Denmark, Lyngby, Denmark; 25 Ecologic Institute, Berlin, Germany; 26 Countryside and Community Research Institute, University of Gloucestershire, Cheltenham, United Kingdom; 27 Universidad Politécnica de Cartagena, Cartagena, Spain; 28 INSERM U1219, EPICENE Team, Bordeaux University, Nouvelle-Aquitaine, France; 29 Institute of Agriculture and Tourism, Department of Agriculture and Nutrition, Poreč, Croatia; 30 Biotechnical Faculty, University of Ljubljana, Ljubljana, Slovenia; 31 Department of Agroecology, Aarhus University, Aarhus, Denmark; 32 Instituto Nacional de Tecnología Agropecuaria—INTA, Buenos Aires, Argentina; PLOS: Public Library of Science, UNITED KINGDOM

## Abstract

Current farm systems rely on the use of Plant Protection Products (PPP) to secure high productivity and control threats to the quality of the crops. However, PPP use may have considerable impacts on human health and the environment. A study protocol is presented aiming to determine the occurrence and levels of PPP residues in plants (crops), animals (livestock), humans and other non-target species (ecosystem representatives) for exposure modelling and impact assessment. To achieve this, we designed a cross-sectional study to compare conventional and organic farm systems across Europe. Environmental and biological samples were/are being/will be collected during the 2021 growing season, at 10 case study sites in Europe covering a range of climate zones and crops. An additional study site in Argentina will inform the impact of PPP use on growing soybean which is an important European protein-source in animal feed. We will study the impact of PPP mixtures using an integrated risk assessment methodology. The fate of PPP in environmental media (soil, water and air) and in the homes of farmers will be monitored. This will be complemented by biomonitoring to estimate PPP uptake by humans and farm animals (cow, goat, sheep and chicken), and by collection of samples from non-target species (earthworms, fish, aquatic and terrestrial macroinvertebrates, bats, and farm cats). We will use data on PPP residues in environmental and biological matrices to estimate exposures by modelling. These exposure estimates together with health and toxicity data will be used to predict the impact of PPP use on environment, plant, animal and human health. The outcome of this study will then be integrated with socio-economic information leading to an overall assessment used to identify transition pathways towards more sustainable plant protection and inform decision makers, practitioners and other stakeholders regarding farming practices and land use policy.

## 1 Introduction

Conventional farms rely strongly on the use of Plant Protection Products (PPP) to secure yields and food safety in crop production and animal husbandry. In Europe between 420,000 and 500,000 tonnes of pesticides—i.e. PPP and are other non-agricultural pesticides—are used annually [1]. Since most of the applied PPP do not reach target species, multiple PPP residues are commonly found in soil, surface water, groundwater, crops, food and feed, animals and humans [2–6]. Fewer residues and lower PPP concentrations are observed in more regulated farming systems such as integrated pest management or organic production [5, 7]. Almost 50% of the active substances approved as PPP in the EU market are bio-accumulative and 25% are persistent in soil [8]. According to the Classification, Labelling and Packaging Regulation [9], 30% of approved PPP have a high acute aquatic toxicity and 28 are suspected carcinogens. Thus, approved substances are potentially harmful to terrestrial and aquatic ecosystems, plants, animals and human (EPAH) health. Data on the distribution of PPP across European agricultural landscapes that account for ecological and environmental variability are however scarce and fragmented. Therefore, there is a need to retrieve the critical data, using harmonised data collection approaches, necessary to deliver an integrated assessment of the overall risk and impact of PPP formulations, residues and their metabolites across the EU. Although a general framework for a more sustainable use of PPP is in place [10], the tools, methods and measures, especially those aimed at a global assessment of impacts and supporting the global sustainable development agenda, are lacking. Transdisciplinary assessments, using a global health approach which include sustainability and cost-benefit analysis (to reduce reliance on PPP while safeguarding the competitiveness of EU agriculture), are necessary to define transition paths to more sustainable use of PPP.

The SPRINT project (on Sustainable Plant Protection Transition, https://sprint-h2020.eu/), funded under the EC program H2020 and launched in September 2020, addresses all these challenges. SPRINT uses a multi-actor global health approach with the following main objectives:

1. Engage with local, regional, national and international stakeholders to identify knowledge needs, and improve awareness of and trust in integrated pesticide risk assessments.
2. Assess PPP component mixtures and their distribution in Environment, Plant, Animal and Humans (EPAH), and related health states in conventional/integrated and organic farming systems.
3. Estimate direct and indirect PPP exposure levels in representative case study sites (CSS).
4. Develop laboratory tests for determining PPP mixture effects.
5. Develop a Global Health Risk Assessment Toolbox for risk and impact assessment of PPP mixtures, linking exposure to health impacts.
6. Assess integrated risks, costs and benefits of PPP use in different farming systems at micro- and macroeconomic levels.
7. Propose transition pathways towards sustainable plant protection, provide policy recommendations, and develop a research agenda.

This manuscript outlines the methods to achieve objectives 2 and 3 of the project, and contributes to objectives 6 and 7. It describes the field procedures used to collect systematic and comparable data in 11 CSS, located across various landscapes, and covering the main EU crops, comprising conventional and organic farm systems. More specifically, this protocol will be used to collect information on: i) PPP component mixtures and their distribution in study populations relevant to EPAH; ii) health parameters of plants, organisms and humans; iii) agronomic management practices and economic data involved in food production; and iv) PPP risk perception and transition options according to different stakeholders.

This protocol offers a comprehensive, transdisciplinary approach to global health assessment. It uses a multi-actor participatory approach to identify more sustainable plant protection practices. This protocol can therefore be of use to others seeking to gather data in a systematic way across areas allowing meaningful integrated assessments. This study protocol description focuses on the design and rationale and provides an overview of the field work (see Supporting Information).

## 2 Overall design and rationale

**Fig 1:**
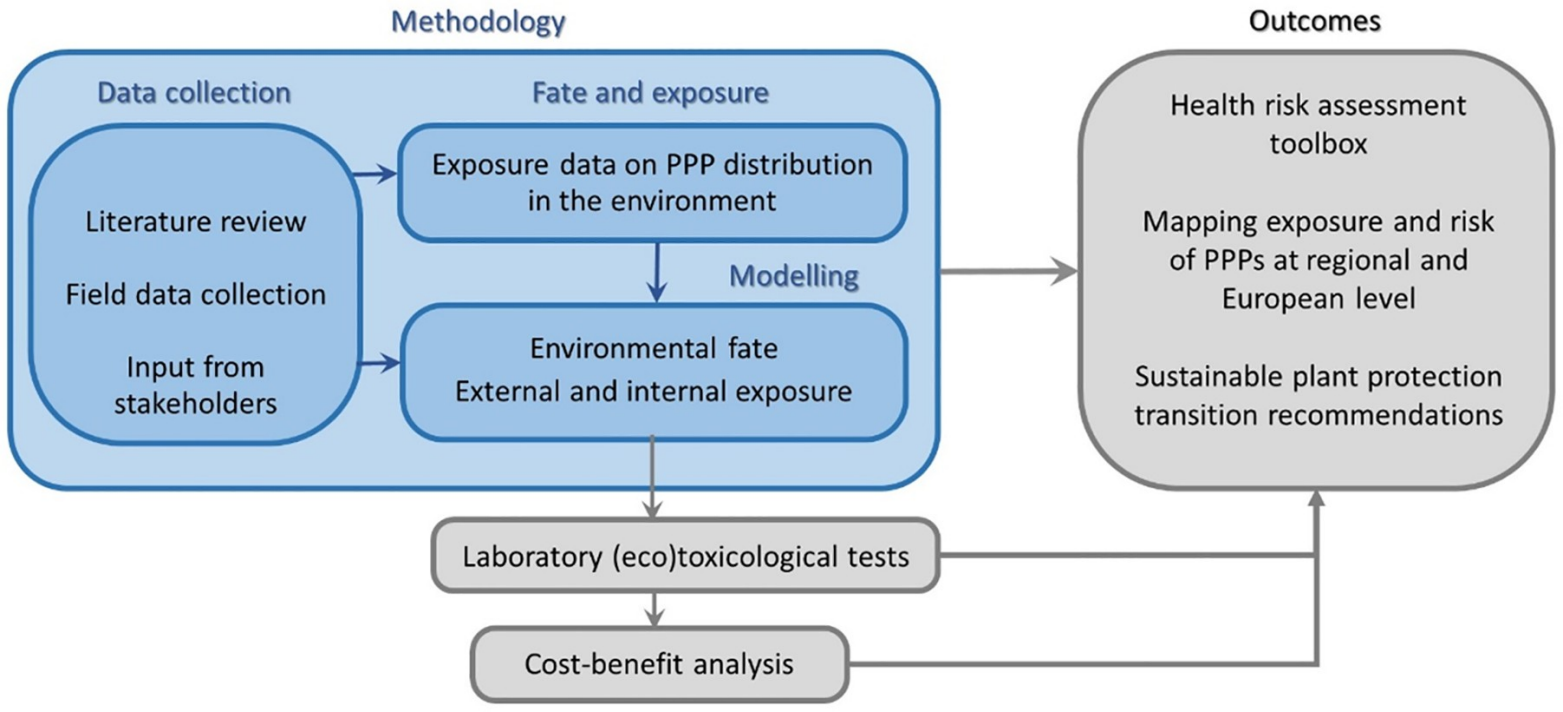
Schematic representation of SPRINT work organization. Only the activities in blue boxes represent field related activities covered in this study protocol.

**Fig 2:**
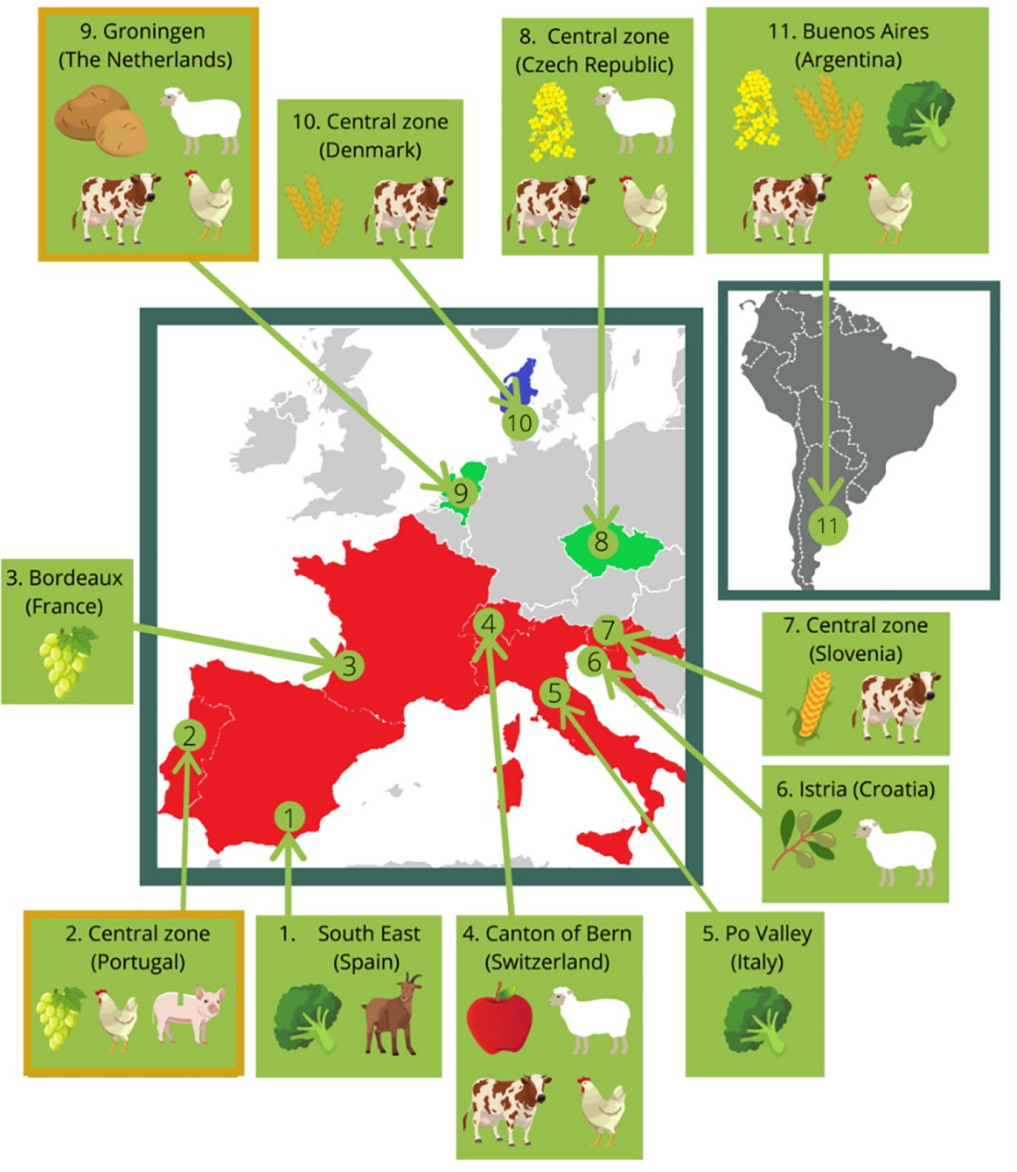
SPRINT Case Study Sites (CSS) location, crops and livestock. Blue on the map = Northern Europe, Green = Central Europe, Red = Southern Europe. Italian and French CSS have no livestock. CSS 2 (Portugal) and CSS 9 (Netherlands) are reference CSS for modelling. At these reference locations, additional water, sediment and air samples will be collected.

This manuscript is organized as a descriptive study protocol that will provide the necessary data for modelling of PPP applications related to EPAH health (*Fig 1*). For this we will use a variety of models that describe the fate of PPP components in the environment and the exposure of EPAH. The field and modelling data will be used for an integrated assessment of the impact of PPP applications on EPAH health. The overall design that best serves this aim is a cross-sectional design at different locations in Europe and one in Argentina (*Fig 2*), covering multiple crops of conventional and organic production. The CSS locations cover the different EU regulatory zones of PPP regulation (North, Central and South), most European climate zones and a selection of the main crop types in the EU: viticulture, horticulture, root crops, fruits trees, olives, cereals and oil seeds. The existence of previous PPP use and monitoring data in the area, and of running/envisioned public discussions on PPP agenda reinforced the inclusion of some CSS. The Netherlands and Portugal—two CSS with contrasting characteristics, and where PPP related projects have been running in the past years—are used as reference CSS for modelling data collection purposes. Argentina was added to the study to include a possible source of indirect contamination coming from soya feed products, which are imported and used in EU conventional farms as livestock feed.

Environmental and biological samples will be collected in 2021 during the growing season. The exact timing of the samples’ collection is aligned to the middle of the growing season for each crop; when we expect the highest diversity and (bio)monitoring levels of PPP. This is also a good period to study impacts on non-target species, as it is a period of high biological activity. Besides the collection of samples, the following information will be collected in each CSS: natural and socio-economic status, farming system, field and crop characteristics, economic performance, perceptions on PPP use and transitions, and health status information. CSS teams received training on study protocol implementation, namely on sample collection and handling procedures, and on interviewing study participants and stakeholders, in order to safeguard quality and comparability of data collected.

The field sampling campaigns will provide new input data for fate and exposure modelling of PPP, but also on EPAH health status, agronomic practices and economic data, and on perceptions regarding PPP use. *Table 1* summarises the covered matrices and the parameters analysed in the field samples. *Table 2* outlines the fate and exposure modelling work, including their output type and use. The relevance of different matrices and populations and data flow is explored in the next sections.

**Table 1:** Overview of matrices and parameters to be measured or inferred at each CSS.

	Matrix	Data to be collected
Environment	Soil	PPP content; microbial composition (microbiome); soil organic matter (SOM); carbon mineralization (C_min_); nitrogen mineralization (N_min_); Phospholipid fatty acid (PLFA) analyses; enzyme activities; functional gene analyses; soil texture, pH and bulk density
	Surface water and sediments	PPP content and total suspended solids in surface water; PPP content, SOM and granulometry in sediment; diversity of benthic macro-invertebrates
	Gas phase and airborne particulate matter	PPP in air and dust outdoor, and in dust indoor
	Plant	PPP content; yield data; pest incidence*, weed infestation*, and diseases*
	Earthworm	PPP; diversity; gut microbiome
	Fish	PPP content in fillet and in liver; gut microbiome
	Bats	PPP content in bat faeces; gut microbiome
	Insects	Biodiversity of ground-dwelling insects and of flying insects
Farm animals	Urine	PPP content; biomarkers
	Faeces	PPP content; microbiome
	Blood	PPP content; biomarkers
	Wristbands	PPP content
	Milk (if applicable)	PPP content
	Feed	PPP content
	-	Reproductivity and diseases*
Human	Urine	PPP content; biomarkers
	Faeces	PPP content; microbiome
	Blood	PPP content; biomarkers
	Wristbands	PPP content
	Nasal swabs	Microbiome
	Food/beverages	PPP content
	-	Reproductivity and diseases*

* Contextual information to be collected by questionnaires.

**Table 2:** Overview of fate and exposure models to be used in CSS, including their output type and use.

Model	Output	Used for	Model verification data
TOXSWA	PPP concentrations in the surface water and in the sediment layer	Aquatic organisms’ exposure	PPP concentration in ditch water and sediment
PEARL	PPP and metabolites concentrations in soil	Soil organisms’ exposure	PPP concentrations in soil
MERLIN-Expo Tool	PPP concentrations in blood and target organs	Animal exposure	PPP concentrations in animal samples
BROWSE (PEARL-OPS, new wind erosion module)	PPP concentrations in ground deposit*	Input for human exposure	PPP concentrations in ground deposit*
	PPP concentrations in air (gaseous and bound to airborne particulates)	Input for human exposure	PPP concentrations in air
OBO-Dustpred	PPP concentrations in indoor house dust	Input for human exposure	PPP concentrations in indoor house dust

* Ground deposit refers to the fraction of spray drift that deposits, either via wet or dry deposition, on the O-Horizon soil layer.

## 3 Populations

**Fig 3:**
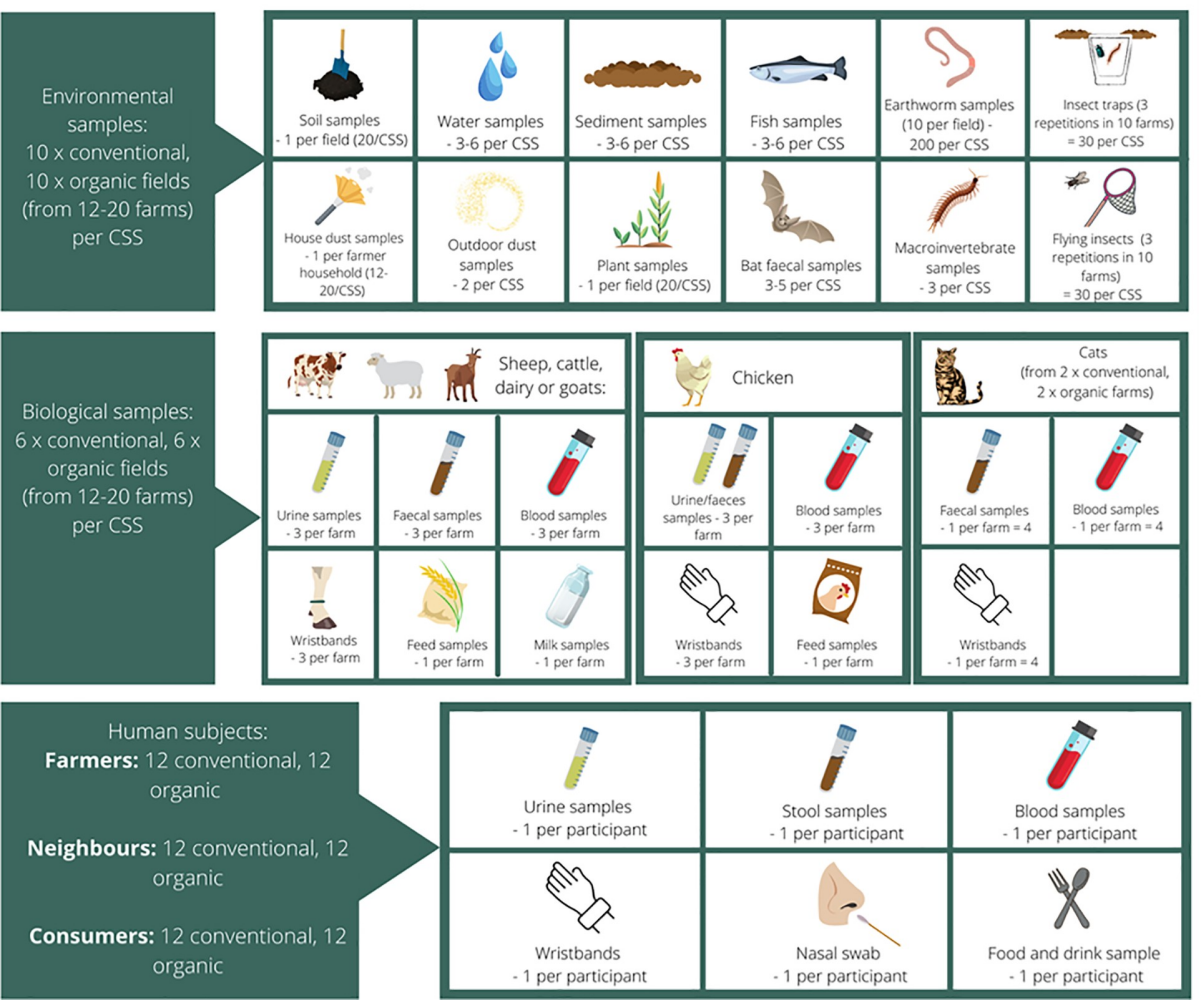
Overview of matrices and populations sampled in SPRINT Case Study Sites (CSS) and respective sampling effort. Each CSS includes 20 fields (10 conventional/integrated and 10 organic). A maximum of two fields are selected per farm; at least 6 organic and 6 conventional farms are selected per CSS.

The study design includes three main populations that are considered recipients of PPP exposure in their own living environment: non-target species, livestock, and humans (*Fig 3*). The non-target species may live on or nearby farmland (e.g., earthworms and fish in downstream freshwater, respectively), or may live further away from agricultural fields but are attracted to the insects near farm buildings and the rural landscape (bats). Livestock may be found in/around the farm buildings, or on the fields around the farm. Humans are divided into three subgroups of different exposure: farmers, neighbours and consumers (see 3.3 for definitions and details).

### 3.1 Non-target species

PPP exposure may affect non-target organisms in numerous ways, often being reported to cause/be associated with impaired behaviour, development and reproduction, or even reduced survival [11]. Accumulation of PPP in resistant or tolerant species may also lead to bio-accumulation. This study focuses on terrestrial non-target organisms living on, or attracted to the farmland, and on aquatic non-target organisms living in water bodies connected or in the vicinity of the agricultural fields. Furthermore, as PPP are closely linked to food security and food safety issues [12], this study focuses also on the main crops in the CSS (*Fig 2*): vineyards, vegetables, root crops, fruit trees, olives, cereals and oil plants.

#### 3.1.1 Soil microorganisms and earthworms

Soil organisms like bacteria, fungi and soil fauna are responsible for nutrient transformation, carbon cycling and degradation of organic pollutants. Depending on the amount and type of PPP, soil organisms and their activities can be positively or negatively affected. In this study, we will measure carbon and nitrogen mineralization, the activities of soil enzymes involved in C-, N- and P-cycling, quantify functional genes involved in PPP degradation, and sequencing soil microbiome (which will give information about the response of bacteria, fungi and archaea to PPP at high taxonomical resolution). PPP are also known to adversely affect and contribute to earthworm populations decrease [13]; the severity and subsistence of PPP effects depends strongly on the type of PPP and earthworm species considered [14]. Given earthworms ecosystem engineers role, in this study we will collect earthworms to assess diversity, PPP content, and gut microbiome.

#### 3.1.2 Fish

PPP may accumulate in aquatic organisms at concentrations much higher than the ones found in the water column [15]. In addition, several substances, including PPP, can sink into sediments and persist for long periods, which can constitute a secondary source of contaminants over time [16]. Hence, fish are considered an effective indicator to assess and monitor the PPP impacts over time and space in aquatic ecosystems [17, 18] as well as to infer the potential risks for human health due to consumption of contaminated fish. In this study, fishes of the most common species per CSS will be caught and used to assess PPP content and microbiome.

#### 3.1.3 Benthic macro-invertebrates

In this study, the macro-invertebrate communities are used as a proxy for aquatic biodiversity impairment. Benthic macro-invertebrates are added to this study to cover the link between exposure and ecological effects at the community level in the aquatic compartment, hence extending the primarily proposed monitoring of exposure, bioaccumulation and effects at the individual level of fish species. Since benthic macro-invertebrates are very sensitive to pollution and linked to calibrated metrics that can accurately reflect the ecological status of the waterbody, they are good indicators of disturbed aquatic ecosystems [19].

#### 3.1.4 Bats

PPP are known to affect bats; next to the potential direct skin contact in their roosts, bats in Europe are indirectly affected via insects, their main food source [20]. Better understanding the relations between the distribution of PPP in ecosystems, food availability and bats health is eminent for their protection. As all bat species are endangered species, sampling in CSS will only consist of the collection of faeces (used also to assess PPP content and microbiome).

#### 3.1.5 Insects

Non-target and beneficial insects are known to be negatively affected by PPP, with for example the effects of neonicotinoid insecticides receiving much attention in the last decade [21]. As insects are susceptible to PPP, insects are considered indicator species to quantify differences in the use of PPP between fields [22, 23]. In this study we will collect ground-dwelling and flying insects, in three 21-day cycles (for flying insects the cycles may be slightly longer depending on weather conditions) to assess biodiversity.

#### 3.1.6 Cat

In the study the cat is preferably a domestic ‘farm’ cat that lives outdoors and to some extent reflects the life of a (wild) predator species. It is supposed that the blood samples from the cat will reflect bio magnification of PPP from prey rodents and birds who in turn feed on left-overs in and around barns and on fields near the farm. Cat results will inform on exposures expected to occur in wild predator species higher up in the food chain.

#### 3.1.7 Crops

By focusing on most crop types in the EU, this study will contribute to PPP productivity and food safety discussions [12, 24]. Different parts of the CSS plants will be tested for PPP–these parts are crop type specific, being selected based on what is used or processed for the final product, or used as food or feed. PPP data will be complemented with crop yield, pest incidence, weed infestation, and plant diseases data.

### 3.2 Livestock

Animals exposed to PPP via dermal, inhalation, oral routes and, if applicable, via lactation, are known to suffer multiple adverse effects. Reproductive effects and infertility are of special concern as they are associated with big economic losses in the livestock industry [25–27]. CSS will focus on the main livestock type(s) in the area, and overall the SPRINT CSS cover cattle, sheep, goats, pigs and poultry. Besides relevance for livestock health assessment, information on PPP in livestock and livestock products are essential to quantify PPP transfer processes from feed to animal, and from animal to human. Different samples are collected from each animal, see section 4.3 and 4.4 for details on sampling methods.

### 3.3 Humans

Farmers, neighbours and consumers are recruited in each CSS to capture a range of different exposure scenarios that cover those defined by the European Food Safety Authority, EFSA [28]. Two exposure patterns are currently used in exposure modelling of PPP by EFSA: consumer and bystander. Consumer is well covered in 3.3.3 but the bystander must be further explored as it can apply to different individuals. Bystanders, i.e. the persons at the periphery of a field where PPP are applied [28], can be farmers (operators or workers) and additional household members of what we would like to refer to as ‘farmer family’, or another type of rural residents with no professional involvement in farming, referred to as ‘neighbour families’.

#### 3.3.1 Farmer families

As mentioned above, two contrasting types of farm(er)s are studied: conventional and organic. The farmer is the central person involved in PPP use decisions; he/she may apply PPP and be therefore prone to occupational exposure. As farmers are often living on or near the farmland, the farmer and farmer family are a relevant target population [29] with a higher exposure likelihood than non-farmer families living in a rural environment [30]. The consumption of agricultural products by farmer families is also considered a potential source of PPP residues’ exposure. These products may be locally produced, imported from EU member states or to some extent also related to products imported from outside the EU.

#### 3.3.2 Neighbour families

Neighbours are those with a main residence adjacent to or at short distance from fields, with no professional involvement in farming. Members of these neighbour families may be exposed to PPP directly by spray drift when they are outside during spray applications. PPP may also disperse as vapours, aerosols or absorbed on the surface of airborne particulate matter that penetrates into the residence [31, 32]. Environmental exposures are then defined as ‘rural background’ and depend on the type of crop and type of farm systems in the region. Tracking back on footwear is another mechanism by which PPP residues may be translocated into the home environment [33, 34]. Again, consumption of agricultural products can be a potential source of PPP residues’ exposure, as some families may buy local products from neighbouring farms.

#### 3.3.3 Consumers

Consumers are citizens living at greater distances from the fields than neighbours, in surrounding villages or towns, and have no professional involvement in farming either. Consumers are considered the reference for exposed persons in terms of environmental exposure. The average consumer is expected to receive most of the PPP exposure through the diet. The mixture of PPP residues they are exposed to is dependent on the composition of their ‘market basket’ that may consist of local products but most likely also products bought from a grocery or supermarket, which may include EU and non-EU imported products. Consumers’ attribution to conventional and organic classes is performed based on the degree of consumption preferences for conventional or organic food.

## 4 Methods of sample collection

### 4.1 Abiotic environment

As mentioned above, five abiotic matrices are covered in the CSS level: soil, water, sediment, outdoor air and home indoor dust. The reasoning on matrices is presented below; details on SPRINT sampling design and storage conditions are summarized in *S2 Table in S1 File*, respectively. The collection of these samples was already finalized in some CSS, while for other CSS it will be initiated soon (this according CSS crops growing seasons timings).

#### 4.1.1 Soil

Soil acts not only as a sink of PPP residues, including persistent residues, but also as a source of PPP residues, when conditions change and via wind and water erosion [35]. 83% of 317 EU agricultural soil samples tested by Silva and co-workers [36] presented PPP residues, 58% presented mixtures.

#### 4.1.2 Water

PPP reach water bodies mainly by surface runoff, leaching and spray drift. Once in water bodies, PPP may impair (drinking) water quality and aquatic life survival and health. For instance, since several of the currently approved PPP active substances have endocrine disruption properties, the reproduction of fish and other aquatic animals is likely affected when exposed to these substances. Furthermore, PPP residues can be transferred from lower to higher trophic levels, with residues being biomagnified through the trophic chain [37].

#### 4.1.3 Sediment

PPP often end up in watercourses, since they are usually intermediate or final recipients of organic contamination [38]. Among the processes inherent in this system, storage mechanisms play a major role in the dissipation of PPP residues, particularly in bottom sediments. PPP sorbed onto suspended particles in the water column are modulated by different parameters such as the texture of the sediment particles and their physico-chemical parameters such as SOM [39].

#### 4.1.4 Gas phase and airborne particulate matter

During application, part of the applied PPP is dispersed into the atmosphere by volatilization or spray drift [40]. The fate of PPP is influenced by their partitioning between the gas phase and the particulate phase while the rate and the extent of the emission after application depends on the physical and chemical properties of the pesticide; the application parameters; the meteorological conditions during and after application; and the characteristics of the target [41]. In addition, fine airborne particulate matter derived by wind erosion from topsoil (usually referred to as ‘dust’) emitted by wind erosion or by tillage activities may act as a transporter for PPP, dispersing it over considerable distance. Once settled, these particulates may act as secondary sources of PPP.

### 4.2 Biotic environment

Seven biotic matrices (i.e. plant/crops, earthworms, fishes, bats, flying insects, ground-dwelling insects and macroinvertebrates) are studied in SPRINT. A justification for the non-target organisms considered is provided in section 3.1. Details on SPRINT sampling design and storage conditions for the selected plants/crops and biota samples are summarized in *S3 Table in S1 File*. Similar to abiotic matrices, the collection of these samples in ongoing or already finalized in some CSS, while for other CSS it will be initiated soon (regarding the CSS crops growing seasons).

### 4.3 Human sample collection

Five sample types were, are being or will be collected from each participant: urine, faeces, blood, wristbands, and nasal swabs. Furthermore, one participant from each subgroup (farmers, non-farmers and consumers) from each CSS will be asked to collect complete duplicate portions of food and beverages consumed during one day. The reasoning for the selected matrices is presented below and the respective sampling design and storage conditions are summarized in *S4 Table in S1 File*. See section 8 ethics, inclusion and exclusion criteria of participants, and privacy protection implications.

#### 4.3.1 Urine

Most PPP active ingredients are readily bio-transformed and appear in urine as conjugated or free metabolites. In addition to these residues, urine will be analysed for a limited number of biochemical effect biomarkers. In this study we will ask the participants to collect a morning void. For density adjustment urinary creatinine will be determined.

#### 4.3.2 Faeces

Faeces collection is a non-invasive method for sampling the microbiome of the gastrointestinal tract. The gut microbiome plays an important role for host health, and can modify environmental chemicals in the intestinal tract, thereby influencing their biological activity, absorbance and distribution in the body. The composition of the microbiome can also be modified by exposure to environmental chemicals, which can have potentially adverse consequences for host health. In addition, PPP and their metabolites, either unabsorbed or excreted in the bile, can be eliminated via faeces. Part of the faeces samples will be analysed for the presence of PPP and their metabolites, and part is collected in tubes containing storage buffer that stabilizes the genetic material of the microbes in the sample, allowing extraction and sequencing of microbial DNA at a later stage.

#### 4.3.3 Blood

The levels of PPP in blood are a primary study outcome and will serve as input to physiologically-based pharmacokinetic (PBPK) modelling of target dose for organs and organ systems where adverse effects may occur. Further, blood is the sample medium of preference for many additional biochemical effect biomarkers (see section 6.3). Collection of a blood sample is done only once and conducted by venepuncture by trained and authorized healthcare professionals according to national and international standards [42]. We will use a vacutainer system with three types of tubes (for plasma separation, serum separation and whole blood) to support the analyses of interest. The blood will be fractionated on the same day, and the resulting material will be frozen until analysis.

#### 4.3.4 Wristbands

The wristband is a low-tech solution for personal passive sampling of chemicals stemming environmental exposure [43]. The silicon material has the capability to absorb pesticides from the environment reflecting direct contact with environmental chemicals, either solid, liquid or gas phase and has been used before to study exposures to pesticides [44]. The wristbands will be worn by farmers, neighbours and consumers continuously for seven days prior to the collection of urine and blood.

#### 4.3.5 Nasal swab

Nasal swabs will be used to sample the microbiome of the upper respiratory tract. Like in the gastrointestinal tract, the nasal microbes are important for human health. Microbial DNA will be extracted from the swabs, allowing the identification of potential shifts in composition and genomic potential of the respiratory tract microbiota.

#### 4.3.6 Food and beverages (meals)

One participant from each subgroup (farmers, non-farmers and consumers) will be asked to collect complete duplicate portions of food and beverages consumed during one day, and to collect all urine (a 24-h sample) on the same day. The duplicate food and beverage portions will be processed and analysed for pre-selected PPP residues and these will be compared with the ones recovered from urine. This information will be used to verify to what extent the diaries and questionnaire data provide an accurate reflection of the dietary intake of PPP.

### 4.4 Livestock and farm animals

For the sampling of animal urine, faeces, blood and wristbands we use similar methods as for humans, with minor adaptations for blood (i.e. reduced amount for chickens and cats), wristbands (i.e. used as ankle straps or attached to the collar in cows and sheep, attached to the collar in goat and cat, or as a diffusive sampler on the ceiling of chicken barns). In livestock assessment, milk (if applicable) and feed samples will also be collected. The reasoning to consider these matrices is presented below and respective sampling design and storage conditions used in SPRINT are summarized in *S5 Table in S1 File*. No nasal swabs will be collected.

#### 4.4.1 Milk

After uptake, PPP are metabolized, excreted via the urine or distributed to internal organs. PPPs are known residues in milk [45]. Usually, the presence of PPP in feed is the main source of their occurrence in the dairy products. Milk is an important source for human exposure since it is a valuable nutritional product in early life and also feedstock for a range of dairy products.

#### 4.4.2 Feed

Feed originates from several crops such as grass, maize, cereals, rape seed and soya beans. It was shown that fodder maize and sorghum retains residues even during harvesting although these were sprayed during early stages of growth. Nag and Raikwar [46] reported feed as an important source of pesticide residue intake by animals.

## 5 PPP component selection

In order to gain insight in (co)occurrence and fate of PPP, environmental media and animal and human samples will be analysed for PPP components (active substances and degradation products and metabolites). For fate studies the most relevant PPP to be measured will be those applied in the crops in the 11 CSSs shortly before the time of sampling. For hazard assessment, knowledge about the occurrence of any (mixture of) pesticide(s) is relevant.

**Fig 4:**
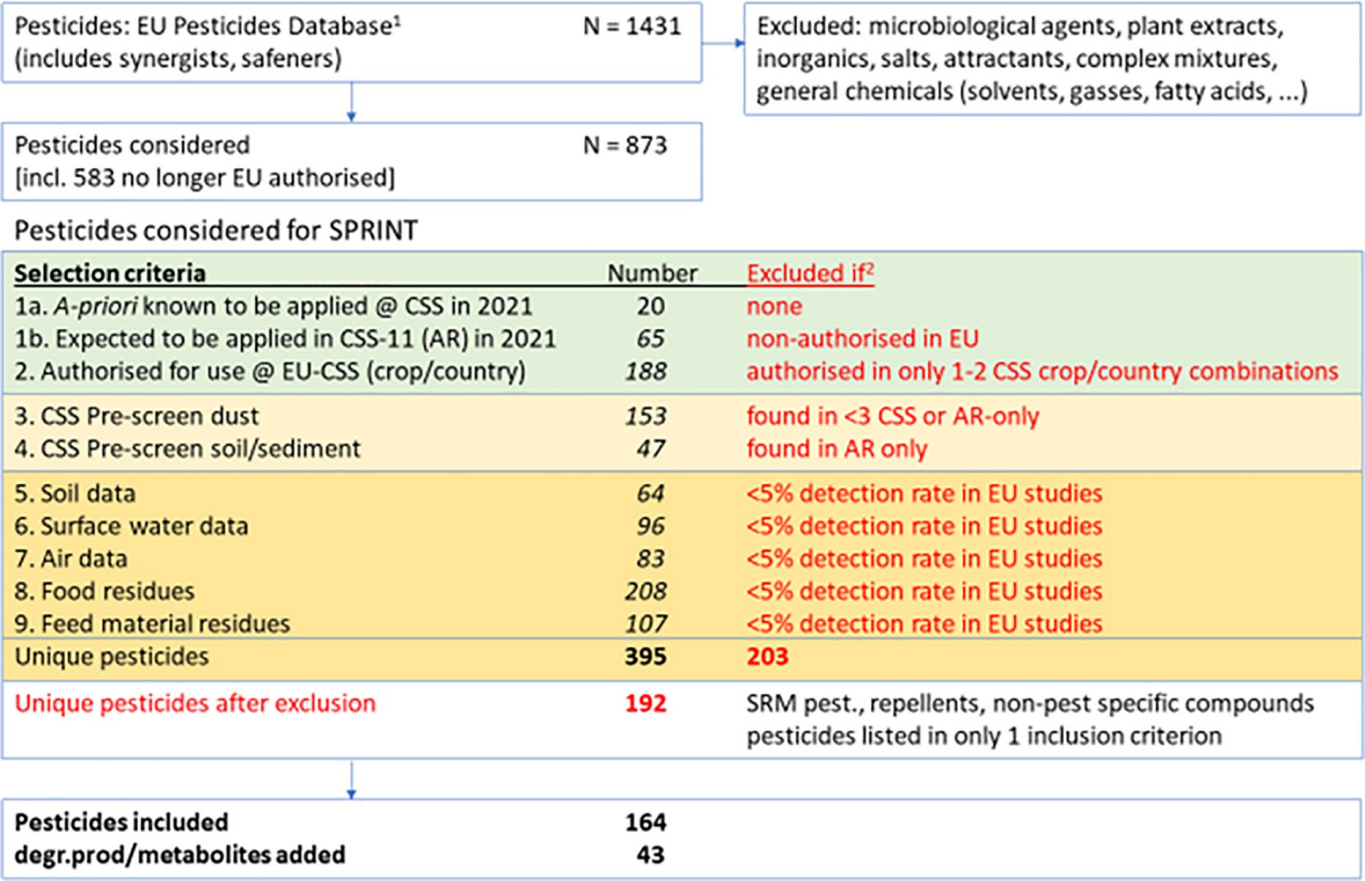
Selection of substances to be included in the analysis of environmental media, human and animal matrices. ^1^Data retrieved in October 2020; ^2^Excluded to bring the total number of substances down to approximately 200, amenable to multi-analyte methods. The reason for exclusion is indicated for each of the 9 selection criteria. SRM = Single Residue Method; AR = Argentina; degr./prod = degradation products.

The EU Pesticides database [47] includes over 1,400 active substances. Although only part of these are authorised (n = 456), many can still be found in the environment or in food and feed and can therefore not be ignored *a priori*. Due to the high number and the wide variety in nature and physical chemical properties, it is inevitable that a selection has to be made for the substances to be included/excluded in the scope of the sample analysis for SPRINT. As a first restriction, only organic molecules are considered. Then, chemicals that can be used for many other purposes than PPP are discarded, leaving a list of 873 chemical substances as a starting point to consider the relevance. The final selection process is summarized in Fig 4 and explained below.

Inclusion in the selection is based on two main criteria: i) known or expected use of PPP in the CSS, ii) data on presence of PPP. Information on PPP used in the upcoming growing season is difficult to obtain at an early stage, so as second best, we compiled the pesticides authorised for the crops/country for each CSS. Recent data on the presence of pesticides at the CSSs were hardly available. To fill this gap, before the actual monitoring study, a pre-screen exercise was done. This involved sampling, in the CSSs region, of dust from the farmer’s premises (household dust and stable dust), soil from agricultural fields, and sediment from water systems. Samples were taken between October 2020 and February 2021, and screened for pesticides using liquid chromatography and gas chromatography with full scan high resolution mass spectrometry [48, 49]. Dust was considered as an archive of pesticides reflecting a longer time period. Soil and sediment samples provided some additional information on more persistent pesticides from the preceding season(s). The occurrence data were complemented with data available from the literature (non-exhaustive) with a focus on more generic EU-wide monitoring data, and more recent sampling dates. Since the project uses a global health approach, all available sources were taken into account, both environmental, and food and feed. Example sources and publications include: Geissen et al. [5], Silva et al. [36] and Riedo et al. [50] for soil; Casado et al. [4] for surface water; Hofmann et al. [2] and Marlier et al. [51] for air; EFSA [52] for food; and WFSR [6] and Mol et al. [53] for feed materials.

Combining the data from all aforementioned sources, the total number of PPP that could be considered relevant for monitoring was almost 400. To reduce this number, for each of the selection criteria (except 1a) an exclusion criterion was set. These criteria are indicated in Fig 4. This resulted in a ‘short-list’ of 192 pesticides. From this list, PPP not amenable to multi-analyte methods were excluded, with the exception of glyphosate and AMPA for which inclusion was already foreseen at the initiation of the project even though a separate analysis method is required for their analysis. This results in a selection of 164 PPP, to which were added 43 relevant degradation products and metabolites (as far as analytical standards are available and amenable to multi-analyte methods).

In order to obtain a consistent data set, and to allow linkage between the various sources and species, the same list of PPP will be analysed in most of the samples, for all CSS. Exceptions are Argentinian and urine samples. For the CSS in Argentina, additional PPP may be added to address the local situation. Some of them may not be authorised for use in the EU. For urine a different scope of analysis will apply. This is because most PPP are not excreted as such, but as their phase I/II metabolites which will considered as the biomarkers of exposure. Their determination is only possible if the main biomarker(s) are known, and if the analytical reference standards are available. For the selection of biomarkers to be analysed in urine, the CSS monitoring data of the environmental samples will be taken into account.

## 6 PPP modelling

### 6.1 Data needs for fate modelling

Exposure estimates will be developed for direct and indirect exposures to PPP mixtures relevant to EPAH health by integrating existing data with new data obtained from CSS, in combination with innovative exposure assessment models. PPP fate in the environment will be assessed by use of existing and/or adapted mathematical models such as PEARL (Pesticide Emission Assessment at Regional and Local scales), and TOXSWA (TOXic substances in Surface WAters). Furthermore, a module will be prepared to compute wind erosion transport, a somehow neglected pathway. An overview on the input data and data needed for modelling validation/verification is provided below:

- Meteorological data: air temperature, rainfall, wind speed and direction, evapotranspiration, solar radiation.
- Air: PPP concentration in gaseous and particulate phase, total suspended particles, wind erosion rate.
- Soil: PPP concentration in soil, field size, soil texture, organic matter content, hydraulic properties, soil erosion rates.
- Crop: PPP concentration in crop, yield, BBCH stage (Biologische Bundesanstalt, Bundessortenamt und CHemische Industrie, scale used to identify the phenological development stages of a range of crop species), leaf index area, crop height.
- Surface water body: size, depth, water flow, distance from field, sediment organic matter content, sediment texture.
- Agronomic management practices: planting time, harvesting time, type of irrigation, physicochemical properties of used PPP, and PPP application schemes.

### 6.2 Data needs for exposure modelling

#### 6.2.1 Human exposure modelling

For human exposure modelling, the OBO exposure module will be used. This module consists of an a priori defined set of equations [29] and includes both human specific input and some more general and specific inputs from CSS. A brief summary of the model input is presented below:

- Human specific input: age, sex, bodyweight, body height, time spent indoors, farmer/neighbour/consumer role.
- General and CSS specific input: season of sampling, output from the environmental modelling to be used in the human exposure assessment, estimates of PPP concentration in different food items, daily lifestyle.

#### 6.2.2 Ecosystems, plants and animal exposure modelling

To estimate animal exposure, we will use the MERLIN-Expo tool [54], that involves an extensive list of inputs; some fixed, some following a probability density function [55], supplied by literature/existing databases and CSS data. For other non-target organisms we will use the MERLIN-Expo tool and the spatio-temporal exposure description by Roeben, Oberdoerster [56]. This approach covers environmental parameters, data on the behaviour of the species, information on toxicokinetics on organisms. There is still not yet a well-established method for the calculation of ecosystems exposure. The use of risk quotients appears a valid approach as it considers estimated environmental concentrations from fate modelling and (no-observed) effect concentrations of the most sensitive species in each ecosystem [57].

### 6.3 Biomarker selection

Following uptake, PPP components are metabolized which leads to internal exposure to metabolites. The cocktail of aforementioned PPP components and their secondary products may be absorbed via different routes of exposure. This uptake triggers a range of responses. Based on a review of the literature (*S1 Table in S1 File*) we have pre-selected exposure and biochemical effect biomarkers that we consider relevant for the potential health impact, that will be assessed in human blood and urine samples across CSS. This list may be updated with new insights.

#### 6.3.1 Exposure biomarkers

As biomarkers of exposure, we will use the PPP components, i.e. active substances, degradation products and metabolites. If possible and if relevant we will also target known co-formulants. The *a priori* selection of these analytes is described in section 8. The results of these exposure biomarker analyses can be compared with the results of PPP residue analysis of environmental samples collected on an individual basis (e.g. diffusive sampling devices like wristbands) and samples collected in the micro-environment (e.g. indoor sample collection in homes of farmers, neighbours and consumers). This can lead to inferences regarding the potential source and route of exposures [30].

#### 6.3.2 Biochemical effect biomarkers

Biomarkers are applied in population-based studies to find support for the involvement of certain molecular or cellular mechanisms to explain adverse effects. However, none of these biomarkers can be used to reflect disease prevalence and incidence directly and, in this study, we will not be collecting data on the occurrence of disease. We include biomarkers of possible value to study associations between exposure and health risk in future large-scale epidemiological studies (*S1 Table in S1 File*). The evidence to support associations between exposure and predictors of effect will be explored by systematic searches of previous studies.

### 6.4 Health status information

Laboratory analysis of PPP and health related components (e.g. microbiome abundance and diversity) will be completed and corroborated by a health status assessment based on interviews (questionnaires are available upon request). These cover mostly:

- Information on overall human health status of CSS participants (demographics, health, medication use, food intake, home environment, working conditions, the use of PPP and personal protective equipment).
- Information on livestock health status (husbandry inventory, feed origin/type, occurrence and manifestation of diseases/malformations/other health problems, use of medication and antibiotics).
- Information on plant health status (pest and disease pressure on plants, yield).

## 7 More contextual information

### 7.1 Agronomic practices and economic data

Farmers will be interviewed before and after the growing season to identify any deviations from planned agronomic practices. Questions cover both natural and human environments, farm and crop characterization, fertilizer and PPP records, and cost and benefits of the assessed crop, including costs of PPP. Information on agronomic management practices and economic input-output data will be used in environmental and economic sustainability assessments as input for farm-level and national-level simulation models. Environmental sustainability models will evaluate both direct impacts on humans and the environment from PPP use as well as life-cycle based impacts of crop protection practices. Empirical analysis of the farm level data involves the determination of trade-offs and synergies between economic performance and PPP risk. Altogether, and in combination with data collected or generated during this study, we will be able to identify innovative and more sustainable plant protection strategies. For one or two selected CSS, an agent-based bio-economic model will then be developed to ex-ante assess the impact of such key strategies on a heterogeneous population of farms, integrating economic and environmental outcome indicators (e.g. farm income, pest management expenditures, quantity of hazardous pesticides applied, pesticide pressure, adoption of integrated or biological pest control, policy costs and revenues).

### 7.2 Perceptions on pesticide use and transitions

To assist farmers in their decision-making towards a transition to the sustainable use of PPP, data are captured through open questions on the levels of current pesticide use, the reasons behind this use, views on conditions that would enable them to reduce pesticide applications, as well as their knowledge and information needs. The latter includes identifying the main types of information they would need when considering taking up a new practice and their preferred sources of information. A slightly adapted version of the questionnaire also asks for perceptions on these questions from neighbours.

## 8 Ethics and privacy protection implications

### 8.1 Ethics approval

The study will be conducted according to the regulations of the European Commission and the current General Data Protection Regulation (GDPR). We will also adhere to the principles of the Declaration of Helsinki (64th World Medical Association General Assembly, Fortaleza, Brazil, October 2013). We will conduct the study with respect for all groups in society regardless of race, ethnicity, religion, and culture, and with respect for and awareness of gender or other significant social differences. To support the submissions of applications to the ethics review boards in each of the 11 CSS, a generic description of the study protocol was prepared with standard solutions for participant information and informed consent. These documents were then translated and adapted to specific requirements in each country. The different ethics related aspects are discussed in more detail below.

#### 8.1.1 Study protocol for ethics approval

A study protocol version was developed specifically for the ethics approval process. This protocol was dedicated to the recruitment of human participants and included details such as inclusion and exclusion criteria for eligibility, the contribution expected from study participants, and information about the potential benefits and burden for each individual participants of joining the study. Both males and females 18 years or older on the day of recruitment can enrol. We will strive for a gender balance within all populations. Individuals who are not able to speak and/or read the local/regional/national language will be excluded from participation. The protocol was submitted to the regional or national ethics committees linked to the CSS, and approved thereafter. Participant recruitment started shortly after the ethics approval.

#### 8.1.2 Personally identifiable information

All personal data will be pseudonymised before any treatment by replacing participant names with a code and protecting all electronic and paper records from unauthorised access (only designated researchers have access to these data). All results will be reported in a strictly non-identifiable format to protect the privacy of every subject. Personally identifiable information will be deleted after completion of the study. The biological specimen and data will be destroyed at the end of the project. The handling of personal data complies with the General Data Protection Regulation (GDPR) and the national legislation on data protection requirements in the country of the CSS.

#### 8.1.3 Participant information and informed consent

Every person invited to participate in the SPRINT study will be informed about the study, before they decide whether to participate or not. Every person who is invited to participate in the SPRINT study gives informed consent before enrolment in the study and can withdraw at any time. For all study outcomes the study leaders will adhere to the right to know and the right not to know as an overarching principle.

### 8.2 Material and data transfer

The material and associated data transferred by the CSS leader to the respective analysing laboratories in line with the terms and conditions of the SPRINT Material and Data Transfer Agreement and the SPRINT Data Policy. Special procedures are in place for transfer of non-EU samples and shipping category B biological substances.

### 8.3 Data use and management

This protocol was prepared as a comprehensive and reference framework to assess the impact of PPP mixture residues, their distribution and exposure across CSS. Data collected with respect to PPP residues distribution in soil will be linked to terrestrial ecosystem health indicators, PPP residues in surface water bodies, and to aquatic ecosystem health. Ecotoxicological tests will be used to investigate these associations in depth. PPP residues detected in animal and human samples will provide first indications on potential linkages between the use of PPP and EPAH systems. Collected data are used as input for modelling and upscaling, and for socio-economic analyses. The main outputs of the fate models are time-resolved PPP concentrations in soil, surface water, sediment, airborne particulate matter, and in the gas phase. These will be further used for the exposure assessment of earthworms, phytoplankton, fishes, animals and humans. Uncertainties in the estimates will be included in the models where possible. CSS and modelling data will be used to study extrapolation of predicted PPP concentrations from local to larger settings, one of the foundations for the development of the global health risk assessment toolbox to be developed by SPRINT. Finally, also innovative agronomic management practices will be identified through CSS data and stakeholder discussions to develop new strategies for reducing PPP use. For one or two selected case(s), using CSS data, stakeholder inputs and agent-based bio-economic modelling, key strategies will be tested in terms of their wider economic and environmental impacts.

In order to manage data efficiently, effectively and securely, a data management plan was drafted in an early stage of the project and updated regularly. All data generated or collected during the SPRINT project will be stored at the SPRINT Nextcloud repository (SPRINT-data.eu) hosted by Netcup (Germany). The database will be secured against unauthorised access by means of a password-protected login process. The project data repository and maintenance of the database will be done by two SPRINT data managers. They will safeguard the data quality, set up required infrastructure for storage and backup, and facilitate data archiving and data sharing. CSS data will be published in peer reviewed open-access scientific journals, and main findings will be disseminated to various target audiences, such as farming communities, rural populations, advisory services, industry, consumers, environmental regulators, and policymakers. First results on PPP determinations should be available around February 2022.

## 9 Potential limitations

This protocol represents a comprehensive yet generic approach to PPP global health assessments. This protocol was submitted to eleven ethics committees linked to each of our CSS. Some committees have requested refinement and asked for further clarifications of the study protocol to meet the respective national/regional ethics requirements. Refinements and some minor changes to the protocol can also be expected during implementation of the proposed sample collection procedures in the field. For example, as CSS teams start their sampling campaign, it is evident that discrepancies in the number of earthworms across fields are to be expected. As in some cases only few earthworms are to be expected in the monoliths, an alternative method (field screen for at least 10 earthworms) will be applied to guarantee enough material for PPP analyses. The implementation of the other parts of the protocol may also have its challenges, e.g., the recruitment of neighbours and consumers due to the potential difficultly to find neighbours that are not farmers or to find people interested in the PPP study outside rural areas, respectively. This might result in slightly more heterogeneous groups than originally envisioned, with some neighbours/consumers being further away from fields than others, and on slightly different numbers of participants across CSS. Finally, the planning of the samples collection campaign might be more challenging than anticipated. In order to collect all samples in the middle of the growing season, and in the shortest period of time possible (to allow links between different matrices data), the agendas of CSS teams, participants, and experts providing services must be perfectly aligned. This becomes specially challenging with atypical cold weather conditions (and consequently delayed PPP applications), and with COVID-19 self-quarantine and isolation situations. In these cases, it is recommended to organize the field work based on closely linked matrices and start with farmers and neighbours samples. Data on consumers, being the reference group in the study, can be collected slightly after the middle of the growing season, if circumstances require. In the reporting of our results, we will discuss potential deviations from this protocol and how it potentially will impact the overall results.

## Supporting information

S1 File (DOCX)

S1 Graphical abstract (PNG)

## Abbreviations

BBCH: Biologische Bundesanstalt, Bundessortenamt und CHemische Industrie (scale used to identify the phenological development stages of a range of crop species)
BROWSE: Bystanders, Residents, Operators and WorkerS Exposure models for plant protection products
C_min_: carbon mineralization
CSS: Case Study Site
EFSA: European Food Safety Authority
EPAH: Environment, Plant, Animal, Human
GDPR: General Data Protection Regulation
IPCHEM: Information Platform for Chemical Monitoring
N_min_: nitrogen mineralization
OBO: Onderzoek Blootstelling Omwonenden (in English—Research on exposure of residents to pesticides in the Netherlands)
OPS: Operational atmospheric transport model for Priority Substances
PBPK: Physiologically Based PharmacoKinetic
PEARL: Pesticide Emission Assessment at Regional and Local scales
PLFA: PhosphoLipid Fatty Acid
PPP: Plant Protection Products
SOM: Soil Organic Matter
SPRINT: Sustainable Plant Protection Transition
SRM: Single Residue Method
TOXSWA: TOXic substances in Surface WAters
