# Supplementary material for: Collection of human and environmental data on pesticide use in Europe and Argentina: Field study protocol for the SPRINT project
Source: PLoS One. 2021 Nov 15;16(11):e0259748. doi: 10.1371/journal.pone.0259748 (PMC8592492; doi:10.1371/journal.pone.0259748)
Supplement: S1 File — (DOCX) [file pone.0259748.s001.docx]

Supporting Information

**Collection of human and environmental data on pesticide use in Europe and Argentina: field study protocol for the SPRINT project**

Vera Silva ^1¶^, Abdallah Alaoui ^2, 3¶^, Vivi Schlünssen ^4, 5^, Anne Vested ^4^, Martien Graumans ^6^, Maurice van Dael ^6^, Marco Trevisan ^7^, Nicoleta Suciu ^7^, Hans Mol ^8^, Karsten Beekmann ^8^, Daniel Figueiredo ^9^, Paula Harkes ^1^, Jakub Hofman ^10^, Ellen Kandeler ^11^, Nelson Abrantes ^12^, Isabel Campos ^12^, María Ángeles Martínez ^13^, Joana Luísa Pereira ^14^, Dirk Goossens ^1^, Juergen Gandrass ^15^, Freya Debler ^15^, Esperanza Huerta Lwanga ^1^, Marlot Jonker ^16^, Frank van Langevelde ^17^, Martin T. Sorensen ^18^, Jerry M. Wells ^19^, Jos Boekhorst ^19^, Anke Huss ^9^, Daniele Mandrioli ^20^, Daria Sgargi ^20^, Paul Nathanail ^21^, Judith Nathanail ^21^, Lucius Tamm ^22^, Peter Fantke ^23^, Jennifer Mark ^22^, Christian Grovermann ^22^, Ana Frelih-Larsen ^24^, Irina Herb ^24^, Charlotte-Anne Chivers ^25^, Jane Mills ^25^, Francisco Alcon ^26^, Josefina Contreras ^26^, Isabelle Baldi ^27^, Igor Pasković ^28^, Glavan Matjaz ^29^, Trine Norgaard ^30^, Virginia Aparicio ^31^, Coen J. Ritsema ^1^, Violette Geissen ^1&^, Paul T.J. Scheepers ^6&*^.

**Affiliations:**

1 – Soil Physics and Land Management Group, Wageningen University & Research, Wageningen, Netherlands.

2 – Institute of Geography, University of Bern, Bern, Switzerland.

3 – Centre for Development and Environment, University of Bern, Bern, Switzerland.

4 – Department of Public Health, Aarhus University, Aarhus, Denmark.

5 – National Research Centre for the Working Environment, Copenhagen, Denmark.

6 – Radboud Institute for Health Sciences, Radboudumc, Nijmegen, Netherlands.

7 – Department for sustainable food process (DISTAS), Università Cattolica del Sacro Cuore, Piacenza, Italy.

8 – Wageningen Food Safety Research, Wageningen, Wageningen University & Research, Netherlands.

9 – Institute for Risk Assessment Sciences, Utrecht University, Utrecht, Netherlands.

10 – Research Centre for Toxic Compounds in the Environment (RECETOX), Faculty of Science, Masaryk University, Brno, Czech Republic.

11– Institute of Soil Science and Land Evaluation, Soil Biology Department, University of Hohenheim, Stuttgart, Germany.

12 – Centre for Environmental and Marine Studies and Department of Environment and Planning, University of Aveiro, Aveiro, Portugal.

13 – Centro de Investigaciones Energéticas, Medioambientales y Tecnológicas – CIEMAT, Madrid, Spain.

14 – Centre for Environmental and Marine Studies and Department of Biology, University of Aveiro, Aveiro, Portugal.

15 – Institute of Coastal Environmental Chemistry, Organic Environmental Chemistry, Helmholtz-Zentrum Hereon, Germany.

16 – Dutch Mammal Society, Nijmegen, Netherlands.

17 – Wildlife Ecology and Conservation Group, Wageningen University & Research, Wageningen, Netherlands.

18 – Department of Animal Science, Aarhus University, Aarhus, Denmark.

19 – Host-Microbe Interactomics, Animal Sciences Group, Wageningen University & Research, Wageningen, Netherlands.

20 – Cesare Maltoni Cancer Research Center, Ramazzini Institute, Bologna, Italy.

21 – Land Quality Management - LQM, Nottingham, United Kingdom.

22 – Research Institute of Organic Agriculture - FIBL, Frick, Switzerland.

23 – Quantitative Sustainability Assessment, Department of Technology, Management and Economics, Technical University of Denmark, Lyngby, Denmark.

24 – Ecologic Institute, Germany.

25 – Countryside and Community Research Institute, University of Gloucestershire, United Kingdom.

26 – Universidad Politécnica de Cartagena, Cartagena, Spain.

27 – INSERM U1219, EPICENE Team, Bordeaux University, Nouvelle-Aquitaine, France.

28 – Institute of Agriculture and Tourism, Department of Agriculture and Nutrition, Poreč, Croatia.

29 – Biotechnical Faculty, University of Ljubljana, Ljubljana, Slovenia.

30 – Department of Agroecology, Aarhus University, Aarhus, Denmark.

31 – Instituto Nacional de Tecnología Agropecuaria - INTA, Buenos Aires, Argentina.

¶ These authors share first authorship; &These authors share last authorship.

*Corresponding author: Paul T.J. Scheepers, E-mail address: [Paul.Scheepers@radboudumc.nl](mailto:Paul.Scheepers@radboudumc.nl).

Contents

[**Abbreviations** 5](#_Toc84500924)

[***Table S1*** - Overview of selected biomarkers to explore associations with PPPs exposure and specific health outcomes. 6](#_Toc84500925)

[***Table S2*** - Sampling and storage details used for environmental abiotic matrices. 7](#_Toc84500926)

[***Table S3*** - Sampling and storage details for environmental biotic matrices. 8](#_Toc84500927)

[***Table S4*** - Sampling and storage details for human related matrices. 9](#_Toc84500928)

[***Table S5 -*** Sampling and storage details used in SPRINT for livestock exclusive matrices. Numbers of samples of urine, faces, blood and wristbands and conditions are similar to those in Table S4 for humans. Blood amounts were reduced to chicken and cats. 10](#_Toc84500929)

[**References** 11](#_Toc84500930)

# **Abbreviations**

AchE Acetylcholinesterase

ALT Alanine aminotransferase

AST Aspartate aminotransferase

BChE Butyryl cholinesterase

BSNE Big Spring Number Eight

CSS Case Study Site

C_min_ Carbon mineralization

DHEAS Dehydroepiandrosterone sulfate

FSH Follicle-stimulating hormone

fT4 Functional Thyroglobulin 4 (biomarker of iodine status)

GGT Gamma-glutamyl transferase

GFAP Glial Fibrillary Acidic Protein

GSH Glutathione

GSSG glutathione disulfide

hsCRP High sensitivity C-reactive protein

ICAM-1 Intercellular Adhesion Molecule 1

IL Interleukin

K2-EDTA Ethylene diamine tetra-acetic acid

KIM-1 Kidney injury molecule-1

LH Luteinizing hormone

NAG N‐acetyl‐β‐d‐glucosaminidas

N_min_ Nitrogen mineralization

PST Plasma Separation Tube

PLFA PhosphoLipid Fatty Acid

PPP Plant Protection Product

SAA Serum amyloid A

s-DHEA Dehydroepiandrosterone sulfate

SHBG Sex hormone-binding globulin

SST Serum Separation Tube

T3 Triiodothyronine (thyroid hormone)

T4 Thyroxine (thyroid hormone)

TNF-α Tumor necrose factor alpha

TSH Thyroid stimulating hormone

VCAM-1 Vascular Cell Adhesion Molecule-1

# ***Table S1*** - Overview of selected biomarkers to explore associations with PPPs exposure and specific health outcomes.

| Health outcome | Biomarkers | Species | Study type | References |
| --- | --- | --- | --- | --- |
| Immune system/ inflammatory status | hsCRP, IL-1β, IL-2, IL-4, IL-5, IL-6, IL-8, IL-10, IL-13 and TNF-α, blood cell counts; SAA, VCAM-1 and ICAM-1 | Human/rat/mouse | Population-based/controlled exposure | (Colosio, 1999) (Corsini et al., 2008; Lee and Choi, 2020) |
| Kidney function | KIIM-1, NAG, urinary electrolytes, creatinine, and urinary proteins | Human | Population-based | (Valcke et al., 2017) |
| Reprotoxic and developmental outcomes | DHEAS, FSH, GGT, LH, s-DHEA, SHBG, testosterone, estradiol, progesterone and cortisol | Human | Population-based | (Bretveld et al., 2006) |
| Liver | ALT, AST, GGT and protein electrophoresis | Human/rat/mouse | Population-based/controlled exposure | (Karami-Mohajeri et al., 2017) |
| Neurotoxicity | AChE | Human | Population-based | (Assis et al., 2018) (Storm et al., 2000) |
|  | BChE | Human | Population-based | (Lockridge and Masson P, 2002) |
|  | GFAP | Rat | Controlled exposure | (Garcia et al., 2002) |
| Thyroid | fT4, T3, T4 and TSH | Human/rat/mouse | Population-based/controlled exposure | (Campos and Freire, 2016; Leemans et al., 2019) |
| Metabolome | Metabolomics | Mouse | Controlled exposure | (Wang and Wu, 2015) |
| Metabolome | Metabolomics | Human | Population-based | (Jobst et al., 2013) (Cariou et al., 2016) |
| Microbiome | Gut and nose microflora | Mouse | Controlled exposure | (Yuan et al., 2019) |
| Oxidative stress | GSSG/GSH | Human | In vitro | (Jabłońska-Trypuć et al., 2017) |

# ***Table S2*** - Sampling and storage details used for environmental abiotic matrices.

| Matrix | Sample amount | Sampling design | Storage conditions |
| --- | --- | --- | --- |
| Soil | ~2000 g | Number of samples: 1 composite sample/field; 20 fields/CSS | 4°C (Cmin, Nmin) or  -20°C (PPP and microbiome, PLFA, enzyme activities, and functional gene analyses) |
|  |  | Depth: 0-5 cm deep (permanent crops)  or 0-20 cm deep (annual crops) |  |
|  |  | Sampling time (NL & PT): at middle and at the end of the growing season, and after harvest |  |
|  |  | Sampling time (other CSS): one time sampling, at middle of the growing season |  |
| Water | 2 L | Number of samples: 1 sample/water body; 3-6 water bodies/CSS | -20°C |
|  |  | Depth: Collected sub-superficially |  |
|  |  | Sampling time (NL & PT): before (1x), during (3x), and end of cropping (1x) and after harvest (1x) |  |
|  |  | Sampling time (other CSS): one time sampling, at middle of the growing season |  |
| Sediment | ~500 g | Number of samples: 1 composite sample/water body; 3-6 water bodies/CSS | -20°C |
|  |  | Depth: Collected at 0 – 10 cm deep |  |
|  |  | Sampling time: same as waters |  |
| Outdoor air/dust | TIEM devices:  Not applicable | Number of samples: 2/CSS - 1 in a Conventional, 1 in an Organic field | -20°C |
|  |  | Height: 150 cm above ground level |  |
|  |  | in the field for 2 months; covering the middle of the growing season |  |
|  | High Volume air samplers:  Not applicable | Number of samples: in 1 NL conventional and 1 PT conventional in field | -20°C |
|  |  | Height: 150 cm above ground level |  |
|  |  | Sampling time: in the field for 1 year, samples collected every 15 days |  |
|  | BSNE passive samplers:  All eroded sediments | Number of samples: in conventional and organic fields, but in NL and PT only | 4°C, in the dark |
|  |  | Height: 25, 50, 75, 100 and 150 cm above ground level |  |
|  |  | Sampling time: same as High Volume air samplers |  |
|  | Frisbee samplers:  All eroded sediments | Number of samples: 1 in conventional and 1 organic fields, but in NL and PT only | 4°C, in the dark |
|  |  | Height: ground level |  |
|  |  | Sampling time: same as High Volume air samplers |  |
| Indoor dust | ~ 100 g | Number of samples: 1 per farmer household; 12-20/CSS  (2 for NL and PT) | -20°C |
|  |  | Sampling time: from 1 month prior to human samples collecting until human samples collection day; in NL and PT it is also collected a 2 to 1 month prior sample; these samples are collected from the participants vacuum cleaner bags. |  |

# ***Table S3*** - Sampling and storage details for environmental biotic matrices.

| Matrix | Sample amount | Sampling design | Storage conditions |
| --- | --- | --- | --- |
| Plant/crop | ~200 g | Number of samples: 1 composite sample/field; 20/CSS | 4°C (seeds, grains) or  -20°C (fruits, vegetables, grapes) |
|  |  | What: sampling part dependent on crop use |  |
|  |  | Sampling time: at harvest time |  |
| Earthworm | all earthworms in the monoliths | Number of samples: two monoliths/field (for diversity & microbiome); 40/CSS | For diversity 70% ethanol; for microbiome -20°C |
|  |  | Sampling time: at middle of the growing season |  |
|  | at least 10  earthworms | Number of samples: 1 composite sample/field (PPP); 20/CSS | depuration for 24h, then -20°C |
|  |  | Depth: 0-25 cm deep |  |
|  |  | Sampling time: at middle of the growing season |  |
| Fish | 5 fishes | Number of samples: 1 composite sample (5 fishes from same species)/water body; 3-6 water bodies/CSS | -20C |
|  |  | Sampling time: at middle of the growing season |  |
| Bat | ~10-25 g | Number of samples: 1 composite sample/roost; 3-5 roost/CSS | -20°C |
|  |  | Sampling time: at middle of the growing season |  |
| Ground-dwelling Insects | all insects in the traps | Number of samples: 1 composite sample (12 pitfall traps in 100m2)/field, in 5 conventional and 5 organic fields/CSS => *3 times => 30 samples/CSS | 70% ethanol |
|  |  | Sampling time: 3 measurements during growing season |  |
| Flying insects | all insects in the net | Number of samples: 1 transect/field, 15 minutes - 150 m transects; in 5 conventional and 5 organic fields/CSS => *3 times => 30 transects/CSS | 70% ethanol |
|  |  | Sampling time: 3 measurements during growing season, ideally same days as Ground-dwelling Insects |  |
| Macro-invertebrates | all insects in the net | Number of samples: 1/water body; in 3 water bodies/CSS, using a 1 mm - 500 μm mesh net | 70% ethanol |
|  |  | Sampling time: at middle of the growing season |  |

# ***Table S4*** - Sampling and storage details for human related matrices.

| Matrix | Sampling amount | Sampling design | Storage conditions |
| --- | --- | --- | --- |
| Urine | ~125 ml | Number of samples: 1/ participant; 72/CSS  First morning void  aliquoting urine on sampling day | -20°C after aliquoting  -80°C one sample as back-up |
|  |  | Sampling time: middle of the growing season |  |
|  | 2-3 L | Number of samples: 1/ participant; 72/CSS  24 h urine  aliquoting urine on sampling day  Sampling time: middle of the growing season | -20°C after aliquoting  -80°C one sample as back-up |
| Faeces | ~25 g | Number of samples: 1 (divided into 5 tubes; 1 scoop per tube)/ participant; 72/CSS  First bowel movement of the day when possible, otherwise option to collect last bowel movement of the day before urine collection (for subjects with infrequent bowel movements)  1 tube for microbiome containing storage medium | -20°C  -80°C one sample as back-up |
|  |  | Sampling time: same day of urine collection |  |
| Blood | ~33 mL | Number of samples: 1 (divided into 9 blood vacutainers (PST, K2-EDTA, SST) - different tubes with additives) / participant; 72/CSS  between 7 and 9 am  by venepuncture, single occasion  fractionation performed <2 hours of collection  PST and SST tubes are centrifuged | -80°C after aliquoting |
|  |  | Sampling time: at same day of urine collection |  |
| Diffusive samplers | Not applicable | Number of samples: 1/ participant; 72/CSS  for 7 consecutive days, prior to day of urine collection | -20°C |
| Nasal swabs | Not applicable | Number of samples: 1/ participant; 72/CSS  nasal swab left nostril  at same day of urine collection | -20°C |
|  |  | Sampling time: at same day of urine collection |  |
| Food/beverages | All during 24 h | Number of samples: 1/ participant*; 6/CSS  * 1 conventional and 1 organic farmer, 1 conventional and 1 organic neighbour, and 1 conventional and 1 organic consumers  Duplicate portion analyses  One day during crop growing season | -20°C |
|  |  | Sampling time: One day during crop growing season |  |

# ***Table S5 -*** Sampling and storage details used in SPRINT for livestock exclusive matrices. Numbers of samples of urine, faces, blood and wristbands and conditions are similar to those in Table S4 for humans (1 sample/animal; 3 animals/farm; 6 farm/CSS). Blood amounts were reduced to chicken and cats.

| Matrix | Sampling amount | Sampling design | Storage conditions |
| --- | --- | --- | --- |
| Milk | 100 ml | Number of samples: 1-3/farm; 6-18/CSS  Collected from the animals (N=3/farm) or from the milk container of the farm (N=1/farm, whichever is possible) | -20°C |
|  |  | Sampling time: at same day of animal urine collection |  |
| Feed | 500 g | Number of samples: 1/farm; 6/CSS | 4°C |
|  |  | Sampling time: at same day of animal urine collection |  |

# **References used in SI**

Assis CRD, Linhares AG, Cabrera MP, Oliveira VM, Silva KCC, Marcuschi M, et al. Erythrocyte acetylcholinesterase as biomarker of pesticide exposure: new and forgotten insights. Environmental Science and Pollution Research 2018; 25: 18364-18376.

Bretveld RW, Thomas CMG, Scheepers PTJ, Zielhuis GA, Roeleveld N. Reproductive Biology and Endocrinology 2006; 4.

Campos É, Freire C. Exposure to non-persistent pesticides and thyroid function: A systematic review of epidemiological evidence. International Journal of Hygiene and Environmental Health 2016; 219: 481-497.

Cariou R, Omer E, Léon A, Dervilly-Pinel G, Le Bizec B. Screening halogenated environmental contaminants in biota based on isotopic pattern and mass defect provided by high resolution mass spectrometry profiling. Analytica Chimica Acta 2016; 936: 130-138.

Colosio C. Immune parameters in biological monitoring of pesticide exposure: current knowledge and perspectives. Toxicology Letters 1999; 108: 285-295.

Corsini E, Liesivuori J, Vergieva T, Van Loveren H, Colosio C. Effects of pesticide exposure on the human immune system. Human & Experimental Toxicology 2008; 27: 671-680.

Garcia SJ, Seidler FJ, Qiao D, Slotkin TA. Chlorpyrifos targets developing glia: effects on glial fibrillary acidic protein. Developmental Brain Research 2002; 133: 151-161.

Jabłońska-Trypuć A, Wołejko E, Wydro U, Butarewicz A. The impact of pesticides on oxidative stress level in human organism and their activity as an endocrine disruptor. Journal of Environmental Science and Health, Part B 2017; 52: 483-494.

Jobst KJ, Shen L, Reiner EJ, Taguchi VY, Helm PA, McCrindle R, et al. The use of mass defect plots for the identification of (novel) halogenated contaminants in the environment. Analytical and Bioanalytical Chemistry 2013; 405: 3289-3297.

Karami-Mohajeri S, Ahmadipour A, Rahimi H-R, Abdollahi M. Adverse effects of organophosphorus pesticides on the liver: a brief summary of four decades of research. Archives of Industrial Hygiene and Toxicology 2017; 68: 261-275.

Lee G-H, Choi K-C. Adverse effects of pesticides on the functions of immune system. Comparative Biochemistry and Physiology Part C: Toxicology & Pharmacology 2020; 235.

Leemans M, Couderq S, Demeneix B, Fini J-B. Pesticides With Potential Thyroid Hormone-Disrupting Effects: A Review of Recent Data. Frontiers in Endocrinology 2019; 10.

Lockridge O, Masson P. Pesticides and susceptible populations: people with butyrylcholinesterase genetic variants may be at risk. Neurotoxicology. 2000 Feb-Apr;21(1-2):113-26. PMID: 10794391.

Storm JE, Rozman KK, Doull J. Occupational exposure limits for 30 organophosphate pesticides based on inhibition of red blood cell acetylcholinesterase. Toxicology 2000; 150: 1-29.

Valcke M, Levasseur M-E, Soares da Silva A, Wesseling C. Pesticide exposures and chronic kidney disease of unknown etiology: an epidemiologic review. Environmental Health 2017; 16.

Wang P, Wu Y-J. Applications of Metabonomics in Pesticide Toxicology. Current Drug Metabolism 2015; 16: 191-199.

Yuan X, Pan Z, Jin C, Ni Y, Fu Z, Jin Y. Gut microbiota: An underestimated and unintended recipient for pesticide-induced toxicity. Chemosphere 2019; 227: 425-434.
