# Supplementary figures and images for: Collection of human and environmental data on pesticide use in Europe and Argentina: Field study protocol for the SPRINT project
Source: PLoS One. 2021 Nov 15;16(11):e0259748. doi: 10.1371/journal.pone.0259748 (PMC8592492; doi:10.1371/journal.pone.0259748)

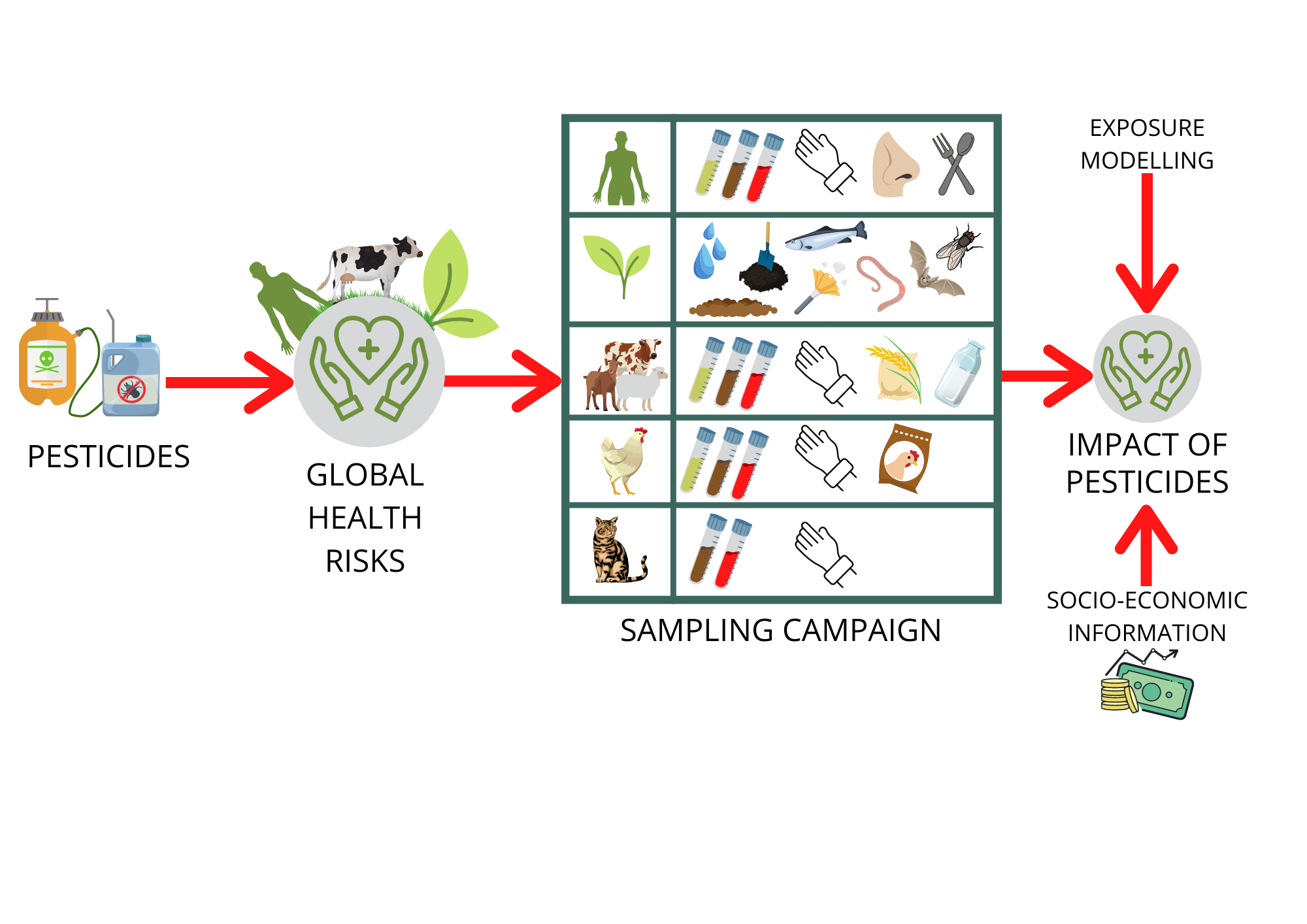

Supplement: S1 Graphical abstract — (PNG) [file pone.0259748.s002.png]
