# Supplementary material for: Collection of human and environmental data on pesticide use in Europe and Argentina: Field study protocol for the SPRINT project
Source: PLoS One. 2021 Nov 15;16(11):e0259748. doi: 10.1371/journal.pone.0259748 (PMC8592492; doi:10.1371/journal.pone.0259748)
Supplement: Peer review history

# Editor report: Decision Letter 0

**DOI:** 10.1371/journal.pone.0259748.r001

**Authors:** Sebastian Shepherd

26 Oct 2021

Collection of human and environmental data on pesticide use in Europe and Argentina: field study protocol for the SPRINT project

PONE-D-21-32372

Dear Dr. SCHEEPERS,

We’re pleased to inform you that your manuscript has been judged scientifically suitable for publication and will be formally accepted for publication once it meets all outstanding technical requirements.

Within one week, you’ll receive an e-mail detailing the required amendments. When these have been addressed, you’ll receive a formal acceptance letter and your manuscript will be scheduled for publication.

An invoice for payment will follow shortly after the formal acceptance. To ensure an efficient process, please log into Editorial Manager at http://www.editorialmanager.com/pone/, click the 'Update My Information' link at the top of the page, and double check that your user information is up-to-date. If you have any billing related questions, please contact our Author Billing department directly at authorbilling@plos.org.

If your institution or institutions have a press office, please notify them about your upcoming paper to help maximize its impact. If they’ll be preparing press materials, please inform our press team as soon as possible -- no later than 48 hours after receiving the formal acceptance. Your manuscript will remain under strict press embargo until 2 pm Eastern Time on the date of publication. For more information, please contact onepress@plos.org.

Kind regards,

Sebastian Shepherd

Associate Editor

PLOS ONE

Journal Requirement:

1. Thank you for submitting your work to PLOS ONE. I am pleased to confirm that having carefully evaluated your manuscript this study protocol has qualified for expedited review as outlined here: https://journals.plos.org/plosone/s/submission-guidelines#loc-study-protocols. 

At this time, we have some very minor copyediting queries/recommendations which require addressing before we can proceed:

Line 368, there are double parentheses at Mol (36). It reads: 'agricultural soil samples tested by Silva, Mol (36)) presented PPP residues, 58% presented'

Line 537, there appears to be an error in grammar for the sentence: ‘Some of them could result not authorised in EU’

Thank you for your attention to these minor edits 

# Editor report: Acceptance letter

**DOI:** 10.1371/journal.pone.0259748.r002

**Authors:** Sebastian Shepherd

4 Nov 2021

PONE-D-21-32372 

Collection of human and environmental data on pesticide use in Europe and Argentina: field study protocol for the SPRINT project 

Dear Dr. Scheepers:

I'm pleased to inform you that your manuscript has been deemed suitable for publication in PLOS ONE. Congratulations! Your manuscript is now with our production department. 

If your institution or institutions have a press office, please let them know about your upcoming paper now to help maximize its impact. If they'll be preparing press materials, please inform our press team within the next 48 hours. Your manuscript will remain under strict press embargo until 2 pm Eastern Time on the date of publication. For more information please contact onepress@plos.org.

If we can help with anything else, please email us at plosone@plos.org. 

Thank you for submitting your work to PLOS ONE and supporting open access. 

Kind regards, 

PLOS ONE Editorial Office Staff

on behalf of

Dr Sebastian Shepherd 

Staff Editor

PLOS ONE
